# Exciting news from *Clinical Proteomics*

**DOI:** 10.1186/1559-0275-8-3

**Published:** 2011-05-31

**Authors:** Daniel W Chan

**Affiliations:** 1 Professor of Pathology, Oncology, Radiology and Urology, Director, Center for Biomarker Discovery and Translation, Johns Hopkins Medical Institutions, Baltimore, MD, USA

## 

As the Editor-in-Chief of *Clinical Proteomics*, I have exciting news to share with you.

*Clinical Proteomics *is entering a new and exciting era of open access publishing. The journal has now been transferred to BioMed Central, an online only open access publisher, which is part of Springer Science and Business Media. Becoming open access and online will result in immediate and free access to all articles, and faster review and publication times. High visibility will result from all content being made accessible *via *web search engines, as well as indexing with PubMed and PubMed Central.

As you know, personalized medicine is expected to revolutionize health care delivery in the next decade. I believe that targeted proteomic diagnostics and therapeutics will be the basis of personalized molecular medicine. Recent advances in proteomic and bioinformatic technologies, such as mass spectrometry and protein microarrays, have been used successfully to detect disease-associated biomarkers in complex biological specimens such as tissues, cell lysates, serum, plasma, and other body fluids. Extensive validation will be needed to translate these biomarkers into targeted clinical use.

*Clinical Proteomics *is in the position to take advantage of these opportunities by providing a scholarly forum for novel scientific research in the field of translational proteomics, with special emphasis on the application of proteomic technology to all aspects of clinical investigations including academic, clinical laboratory, pharmaceutical and diagnostic industries.

We have assembled a strong editorial team of leading scientists in the field of clinical proteomics including Associate Editors Mark Baker, PhD (Sydney, Australia), Robert J. Cotter, PhD (Baltimore, MD, USA), Dennis Hochstrasser, MD (Geneva, Switzerland), Gil Omenn, MD, Ph.D (Ann Arbor, MI, USA) and Hui Zhang, PhD (Baltimore, MD, USA) as well as Editorial Board Members who are outstanding scientists and clinicians in the field of clinical proteomics. I fully expect that *Clinical Proteomics *will be a truly international journal providing an authoritative forum for this field. I hope that you would consider submitting your work to this exciting journal in its new format - *Clinical Proteomics*.

